# Final Disposition and Quality Auditing of the Rehabilitation Process in Wild Raptors Admitted to a Wildlife Rehabilitation Centre in Catalonia, Spain, during a Twelve Year Period (1995–2007)

**DOI:** 10.1371/journal.pone.0060242

**Published:** 2013-04-17

**Authors:** Rafael A. Molina-López, Jordi Casal, Laila Darwich

**Affiliations:** 1 Centre de Fauna Salvatge de Torreferrussa, Catalan Wildlife-Service-Forestal Catalana, Santa Perpètua de la Mogoda, Barcelona, Spain; 2 Departament de Sanitat i Anatomia Animals, Facultat de Veterinària, Universitat Autònoma de Barcelona, Cerdanyola del Vallès, Barcelona, Spain; 3 Centre de Recerca en Sanitat Animal (CReSA), UAB-IRTA, Campus Universitat Autònoma de Barcelona, Cerdanyola del Vallès, Barcelona, Spain; University of Georgia, United States of America

## Abstract

**Background:**

Variability in reporting and classification methods in previous published data of the final dispositions in the rehabilitation of wild raptors makes use of this data limited in trying to audit the quality of the rehabilitation process. Crude as well as stratified disposition rates are needed if quality auditing of the rehabilitation process is to be adequately performed.

**Methodology:**

Final dispositions of 6221 hospitalized wild raptors admitted at a wildlife rehabilitation centre (WRC) of Catalonia during 1995–2007 were analyzed. These dispositions were calculated as the euthanasia (Er), unassisted mortality (Mr), release (Rr) and captivity rates (Cr)., time to death (Td) for dead and euthanized raptors, and length of stay for released (Tr) raptors was estimated. Stratified analyses by main causes of admission and clinical signs were performed.

**Results:**

The disposition for the total population were: Er  = 30.6%, Mr = 19.1%, Rr  = 47.2%, and Cr  = 3%. By main causes of admission, Er was higher in the trauma category (34.2%), whereas Mr was found similar between trauma (37.4%) and non-trauma categories (34.8%). The highest Rr was observed for the orphaned group (77.9%). Furthermore, Cr was low in all the categories (<4%). By clinical signs, the highest Er was found in animals suffering musculoskeletal (37.9%) or skin (32.3%) lesions; Mr was high in infectious/parasitic diseases (66.7%) and in case of neurological symptoms (64.5%). The euthanized birds had a median Td  = 1 day (P_10_ = 0-P_90_ = 59) for both trauma and non-trauma categories, and Td  = 36 days for the orphaned young group (P_10_ = 0; P_90_ = 596). The median Td in the unassisted dead birds was 2 days for all the categories (P_10_ = 0-P_90_ = 31). Finally, the median Tr in the centre was variable among categories.

**Conclusions/Significance:**

Reporting of final dispositions in wildlife rehabilitation should include the crude and stratified rates (Er, Mr, Rr, and Cr), by causes and clinical presentation, as well as Td and Tr, to allow meaningful auditing of the rehabilitation process quality.

## Introduction

Rehabilitation of wild raptors is a complex process that includes both veterinary care of the injured bird and physical recovery and reconditioning of this animal for subsequent release in the wild [1]. The direct benefits derived from the recovery of wild birds could be summarized in several aspects: the improvement of the welfare of the individual animal, the reinforcement of the natural population after the release, especially in endangered species or long-lived birds, the identification of the causes of morbidity and mortality, and the regulatory changes implemented as a consequence of determining human influences and causes of admission [2], [3].

Data published from wildlife rehabilitation centres (WRC) have been mainly focused on the causes of admission [4]–[7], on the investigation of some specific infectious or parasitic diseases and toxicoses [8]–[10] or on the establishment of bio-pathological reference values [11]. On the other hand, the final dispositions of the rehabilitation cases are commonly summarized or briefly described [12]–[14], but a stratified analysis by causes of the final disposition is rarely reported. This kind of analysis is crucial for building an evidence base for wildlife rehabilitation medicine and management.

Quality assessment is one of the strategic elements for the improvement and transformation of the modern human health system [15]. Outcomes research is an essential part of the quality control process, and quality indicators of medical performance have been defined by consensus in order to determine the quality of care in a measurable way [16], [17]. In wildlife medicine, some clinical practice guidelines have been published which deal with welfare rehabilitation standards [18] and pre-release health screening protocols [19] but no quality indicators of the rehabilitation process have been defined.

The main objective of the present study was to analyze the outcomes of the rehabilitation of wild raptors in a WRC, adopting the four categories of the final disposition, the time until death and the length of stay as indicators of the quality audit of the rehabilitation process before release back to the wild.

## Materials and Methods

### Study design and animals

A retrospective study was performed using the original medical records of birds of prey admitted at the Wildlife Rehabilitation Centre (WRC) of Torreferrussa from 1995 to 2007. The centre is under the direction of the governmental Catalan Wildlife-Service. Samples were collected in compliance with the Ethical Principles in animal research guidelines in wildlife rehabilitation centres. The rehabilitation centres directly depend on the individual regional government wildlife services in Spain. Management and protocols were established according to the guidelines approved by each regional government according to legislation [20]. Animals that had to be euthanized for animal welfare reasons were administrated barbiturates by intravenous injection.

### Definition of variables

Overall data about species, gender, age, date of admission, date of death or release, and primary cause of admission were included in the analyses. Classification of primary morbidity causes, criteria for sexing and ageing, as well as the geographical and demographical characteristics of the population were the same as those reported in a previous study [7].

The final disposition was divided into four categories adapted from Cooper (1987) [21]: euthanized animals (based on poor quality of life, or poor prognosis for survival on return to the wild), dead animals (with no human intervention), animals returned to the wild and permanently captive non-releasable animals (due to their poor prognosis of survivability in wilderness). The final dispositions were calculated by dividing the number of cases of each category by the total number of admissions in a given period of time; as a result, all four categories were expressed as rates: euthanasia rate (E_r_), unassisted mortality rate (M_r_), release rate (R_r_), and captivity rate (C_r_). In addition, R_r_ was analysed taking into account the season of admission and the season of release.

The final disposition was first analyzed based on the primary cause of admission grouped as trauma, non-trauma and orphaned young categories. It was then analyzed according to the main clinical signs of the animals at the time of the admission. This clinical presentation was based on the International Statistical Classification of Diseases and Related Health Problems-ICD-10 (WHO, 2004) [22] but adapting the categories to wildlife medicine. We have adopted a single-condition morbidity analysis in which the main condition was defined as the primary condition responsible for the patient's need for treatment or investigation. If there was more than one such condition, the one held most responsible for the greatest use of resources was selected. If no diagnosis was made, the main symptom, abnormal finding or problem was selected as the main condition. In this line, the initial signs were divided into the following categories: apparently healthy animals, infectious/parasitic diseases, endocrine/nutritional/metabolic diseases, behavioural abnormalities (imprinted or tame), eye and adnexa problems, skin and subcutaneous conditions, alterations in the different systems (nervous, respiratory, digestive and musculoskeletal), traumatic signs not classified in any of the previous categories, and others which included birds with different clinical signs not classified in the above categories. In order to minimize overlapping between diagnostic categories, the infectious/parasitic diseases category included all those diseases generally recognized as communicable or transmissible, despite the affected system.

Additional parameters such as time until death (T_d_; difference between the date of admission and the date of the death) for euthanized and for dead animals, and length of stay in the centre for the released raptors (T_r_; difference between the date of admission and the release date) were also evaluated. In order to study the cases with longest T_d_, the percentiles 10 (P_10_), 75 (P_75_) and 90 (P_90_) of this variable were selected as a cut-off point.

Quality indicators of the rehabilitation process conducted at the centre were evaluated based on different outcome variables following guidelines used in human medicine [23], [24]. The main indicators adopted in our work were the four categories of the final disposition, the time until death (T_d_) and the length of stay at the centre for the released raptors (T_r_).

### Statistical analysis

Descriptive statistics, normality test and inferential analyses were done at 95% confidence levels with SPSS Advanced Models ™ 15.0 (SPSS Inc. 233 South Wacker Drive, 11th Floor Chicago, IL 60606-6412). Median (P_50_). Percentiles 10, 75 and 90 (P_10;_ P_75;_ P_90_) were provided for the descriptive analysis of the dispositions T_d_ and T_r_. Comparisons of the median were evaluated using the U-Mann-Whitney and Kruskal-Wallis test. Chi-square (χ^2^) or Fisher exact tests were used for comparisons between the E_r_, M_r_, R_r_ and C_r_ and sex, age and order co-variables.

In order to compare the differences along the period of study of the final disposition categories, a ratio between the number of dispositions and the total number of cases per year was estimated. A linear regression model was used to estimate the trend of the dispositions during the period of study according to the main cause of admission categories and the order.

## Results

### Descriptive analyses of the total population

During a period of twelve years (from 1995 to 2007), a total of 7553 raptor admissions were reported at the WRC. After a critical review of all the admissions, 1332 cases were excluded for not fulfilling the inclusion criteria (739 cases were admitted dead and 593 cases included captive birds, captive-borne or falconry birds). Thus, the final population of this study was 6221 individuals distributed in the following orders: 3241 Strigiformes and 2980 Falconiformes.

The age distribution demonstrated that 46.3% (2884/6221) of birds were within their first year of age, 32.3% (2009/6221) were >1 calendar year and 21.3% (1328/6221) were of unknown age. Most of the animals, 59.4% (n = 3695), were classified as undetermined gender, 21.9% (n = 1363) of raptors were sexed as female and 18.7% (n = 1163) as males.

**Figure 1:**
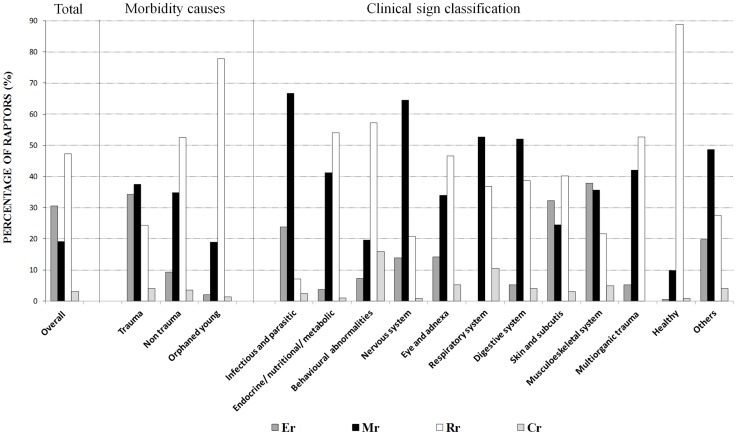
Resolution rates of euthanized (Er), dead (Mr), released (Rr) and captive (Cr) raptors relative to the overall population, the principal cause of the admission and the clinical signs.

A crude analysis of the final disposition of the total raptor population showed the following rates: E_r_ = 30.6% (1903/6221), M_r_ = 19.1% (1191/6221), R_r_ = 47.2% (2939/6221), C_r_ = 3% (188/6221) (Fig. 1).

### Rehabilitation final dispositions by causes of admission

Stratifying by the primary cause of admission, 49.7% (3092/6221) of birds were classified into the trauma category, 15.7% (976/6221) in the non-trauma and 34.6% (2152/6221) in the orphaned young category. The euthanasia rate was notably higher in the trauma category (34.2%) compared to the non-trauma (9.2%) or orphaned young (2%) (Fig. 1), and mainly due to those cases related to electrocution and collisions with power lines (Table 1). The unassisted mortality rate was similar in both trauma (37.4%) and non-trauma (34.8%) categories but lower in the orphaned young (18.9%). Within the traumatic causes, animals found in traps (52.6%), and collisions with vehicles (46.5%) or fences (47.8%) presented the highest unassisted mortality rate. In the non-traumatic causes, infectious/parasitic diseases had the highest rate of mortality (70%). The release rate was significantly higher in the orphaned young (77.9%) and in non-trauma (52.5%) categories compared to the trauma category (24.3%). In the last category, birds who suffered collision with buildings had the best rates of release compared to the other traumatic causes. Finally, low rates of captivity were found in the three categories (4.1% trauma, 3.5% non-trauma) and particularly in the orphaned young birds (1.3%) (Table 1).

**Table 1:** Description of the number and percentage of raptor cases according to their final disposition and the cause of admission at the wildlife rehabilitation centre.

Primary cause	Total	Euthanasia		Mortality		Release		Captivity	
Categories	N	n	Rate (%)	N	Rate (%)	n	Rate (%)	n	Rate (%)
Trauma	3092	1058	34.2	1157	37.4	750	24.3	127	4.1
Unknown trauma	1691	560	33.1	658	38.9	385	22.8	88	5.2
Gunshot	627	183	29.2	210	33.5	207	33.0	27	4.3
Vehicles	471	136	28.9	219	46.5	108	22.9	8	1.7
Electrocution	197	162	82.2	30	15.2	2	1.0	3	1.5
Building	52	3	5.8	17	32.7	32	61.5	0	0.0
Fences	23	9	39.1	11	47.8	3	13.0	0	0.0
Power lines	9	5	55.6	2	22.2	2	22.2	0	0.0
Trap	19	0	0.0	10	52.6	8	42.1	1	5.3
Predation	3	0	0.0	0	0.0	3	100.0	0	0.0

Non-trauma	976	90	9.2	340	34.8	512	52.5	34	3.5
Fortuity*	346	8	2.3	99	28.6	235	67.9	4	1.2
Undetermined	165	27	16.4	71	43.0	63	38.2	4	2.4
Metabolic/nutritional	223	27	12.1	95	42.6	94	42.2	7	3.1
Captivity	156	13	8.3	27	17.3	100	64.1	16	10.3
Infectious parasitic	84	15	17.9	46	54.8	20	23.8	3	3.6
Toxicoses	2	0	0.0	2	100.0	0	0.0	0	0.0

Orphaned young	2153	43	2.0	406	18.9	1677	77.9	27	1.3
									

* Fortuity includes all raptors found in manure heaps, bad weather conditions, etc, as previously defined by Molina-Lopez et al. (2011).

In the subgroup of animals with known sex and age, the unassisted mortality rate was higher in males than in females, in both non-trauma (χ^2^ = 6.6; p = 0.0098) and orphaned young (χ^2^ = 15.8; p = 0.003) categories.

### Rehabilitation final dispositions by clinical signs

The euthanasia rate (E_r_) was higher in those animals suffering lesions at the skin level (32.3%), mostly affected by extensive wounds and electric burns, or at the musculoskeletal system, basically due to fractures and luxations (37.9%) (Fig. 1). By contrast, E_r_ was very low in adults presenting endocrine/nutritional/metabolic disorders (3.7%) and digestive disorders (5.3%). The unassisted mortality rate (Mr) was elevated in raptors with infectious/parasitic diseases (66.7%), mainly trichomoniasis, or with neurological symptoms like depression, ataxia and paralysis (64.5%). The highest rate of release was observed in the apparently healthy animals (88.9%), mostly represented by young orphaned birds and birds belonging to the fortuity category, including birds found inside buildings or other human structures. The R_r_ was also high for animals with behavioural abnormalities (57.3%) and in animals in the endocrine/nutritional/metabolic (54.1%) category when this comprised birds with low body condition and weakness as main general symptoms. Finally, the captivity rate was elevated in those animals with behavioural abnormalities (15.9%) and respiratory distress (10.5%) (Table 2).

**Table 2:** Description of the number and percentage of raptor cases according to the final disposition and clinical signs presented at the admission at the wildlife rehabilitation centre.

Primary clinical signs	Total	Euthanasia		Mortality		Release		Captivity	
	N	n	Rate (%)	n	Rate (%)	n	Rate (%)	n	Rate (%)
Infectious and parasitic	42	10	23.8	28	66.7	3	7.1	1	2.4
Endocrine/nutritional/metabolic	862	32	3.7	355	41.2	466	54.1	9	1.0
Behavioural abnormalities	82	6	7.3	16	19.5	47	57.3	13	15.9
Nervous system	324	45	13.9	209	64.5	67	20.7	3	0.9
Eye and adnexa	206	29	14.1	70	34.0	96	46.6	11	5.3
Respiratory system	19	0	0.0	10	52.6	7	36.8	2	10.5
Digestive system	75	4	5.3	39	52.0	29	38.7	3	4.0
Skin and subcutis	679	219	32.3	166	24.4	273	40.2	21	3.1
Musculoeskeletal system	2110	799	37.9	751	35.6	456	21.6	104	4.9
Multi-organic trauma	19	1	5.3	8	42.1	10	52.6	0	0.0
Healthy	1610	8	0.5	157	9.8	1432	88.9	13	0.8
Others*	193	38	19.7	94	48.7	53	27.5	8	4.1

* Included all cases with other clinical signs not classified in any of the described categories.

### Additional parameters: time until death and length of stay at the centre

The group of euthanized birds had a median T_d = _1 day (P_10_ = 0; P_90_ = 59) for the trauma (P_10_ = 0; P_90_ = 41) and non-trauma (P_10_ = 0; P_90_ = 171) categories, and T_d = _36 days for the orphaned young group (P_10_ = 0; P_90_ = 596) (Table 3). Interestingly, the median T_d_ in the dead birds was 2 days for all the categories (P_10_ = 0; P_90_ = 31). On the other hand, the median time of stay in the centre was highly variable among categories, presenting the trauma the longest times (T_r_ = 115) compared to non-trauma (T_r_ = 58) and orphaned young (T_r_ = 59) groups (Table 3).

**Table 3:** Statistical descriptive of time that animals were keep in the rehabilitation centre until the final disposition.

	Time (days) from admission to final disposition											
	Euthanasia rate				Unassisted Mortality rate				Release rate			
Admission Causes	P_10_	P_50_	P_75_	P_90_	P_10_	P_50_	P_75_	P_90_	P_10_	P_50_	P_75_	P_90_
**Trauma**	0	1	7	41	0	2	5	26	24	115	265	443
Unknown trauma	0	1	7	57	0	2	5	27	24	94	240	416
Gunshot	0	2	22	82	0	3	7	74	66	207	320	621
Vehicles	0	1	10	28	0	2	5	15	14	95	239	485
Electrocution	0	0	1	4	0	2	4	7	N/A	N/A		N/A
Building	0	0	0	0	1	2	6	18	1	45	133	241
Fences	0	1	3	0	1	2	7	477	1	22	Na	N/A
Trap	0	N/A	N/A	N/A	N/A	8	11	15	5	15	148	N/A
**Non-trauma**	0	1	25	171	0	2	5	16	2	58	163	372
Fortuity	0	0	3	298	0	2	4	25	1	37	116	311
Undetermined	0	0	2	156	0	1	3	8	7	51	128	393
Metabolic/nutritional	0	1	25	96	0	2	5	18	11	63	110	280
Captivity	0	16	119	399	0	2	13	68	21	158	320	516
Infectious/parasitic	0	8	19	138	0	2	6	13	30	60	108	372
**Orphaned young**	0	36	187	596	0	2	14	51	18	59	87	179

P_10_, P_50_, P_75_, P_90_: percentiles 10, 50 (or median), 75 and 90; N/A, not applicable (just one case).

Taking into account the season of the admission because it is of relevance for the decision of approving the release of rehabilitated animals, the median T_r_ was statistically different among seasons (χ^2^ = 269.933; p<0,001), with raptors admitted in spring presenting stays of 85 days (P_10_ = 12; P_90_ = 296), 53 days (P_10_ = 16; P_90_ = 212) if admitted in summer, 113 days (P_10_ = 10; P_90_ = 386) if admitted in autumn and 130.5 days (P_10_ = 23; P_90_ = 418) if admitted in winter.

### Time evolution of dispositions along the study period

No statistically significant differences were observed among the final dispositions during the 12 years of the study in the overall group. However, in the traumatic category, a significant decrease in the unassisted mortality rate was observed (B = −0.12; p = 0.035).

## Discussion

Historically, wildlife programs were developed as a consequence of the concern of modern society with both animal welfare and the negative impact of human activities in wildlife population. Rehabilitation of birds of prey and owls has led to the development of many of these programs due to the sensitivity of wild birds to human threats, the unfavourable status of many species and the public interest in these predators [25].

A detailed description of primary causes of admission has been thoroughly reported [26] and welfare and general guidelines for rehabilitation of wild raptors are available [18]. However, the approach to the quality of audit in wildlife rehabilitation is poorly reported. In human medicine, quality indicators of the dispositions are employed to assess and improve the quality of care in many healthcare settings [27]. The data presented in the current study report the crude and stratified dispositions rates by cause and clinical entities, but also the time until death and the length of stay. All six parameters have been considered as quality indicators as a baseline for a quality audit.

From the data it is evident that less than half of raptors admitted to rehabilitation in Catalonia were successfully released. 52.8% of raptor admissions resulted in euthanasia, mortality or permanent captivity. Only 47.2% of birds were successfully returned to the wild. Nevertheless, an estimation of the final dispositions based on the main causes of admission or the clinical entities is essential in order to compare the results. The most simplistic and realistic classification is that consisting of two groups: 1) healthy young birds requiring rearing, 2) injured and ill birds, including those that have been kept illegally in captivity. Orphaned young birds represent an important part of the admissions to the WRC [28], usually concentrated in a short period of time and resulting in filling of rehabilitation facilities to maximum capacity and needing labour intensive care. Moreover, many of the birds are likely not true orphans, but because they are easily found by humans are brought to the WRC [29] and are apparently in good overall health. The proportion of releases in this group is high, and this influences the overall dispositions and results.

Literature on the dispositions of bird of prey rehabilitation is variable, making comparison between studies difficult. Most studies emphasise the release rate [30] as the main outcome, but overall causes are also frequently estimated [13], [31]. In fact, two basic dispositions could be considered: releases and non-releases, including death, euthanasia and captivity of non-releasable birds. In the authors' opinion, the four categories (release, unassisted death, euthanasia and permanent captivity) should be analysed individually as a basic assessment of the quality indicators of the rehabilitation process, due to their different biological and management implications.

Euthanasia is an essential option in all wildlife rehabilitation, based on both animal welfare and optimization of economical resources [1], [32]. However, beyond the situations in which the rehabilitation of the bird is not a viable option and euthanasia is the most appropriate disposition, legal policies preclude the final disposition of a bird of prey in some countries [33]. In our study, the overall rate of euthanasia was 30.6%, and the highest values were found in the trauma category (34.2%) mainly due to electrocutions and collisions with power lines. In our experience these animals frequently cannot be rehabilitated for release due to the severity of their injuries.

Mortality rate has been used as a quality indicator parameter in human medicine [34]. Unfortunately, in wildlife rehabilitation this parameter has been variably reported in most studies without defining criteria, making the comparison of results difficult. In some studies the mortality rate includes the proportion of deaths as well as the proportion of euthanized animals while others do not [13], [35]. This approach may lead to overestimations of the actual rate of non-human intervention results. In our opinion, unassisted mortality rate and proportion of euthanized should be estimated separately and included in the general disposition report.

Our data demonstrated a similar rate of mortality for trauma (37.4%) and non-trauma (34.8%) cases. In the non-traumatic group, the higher M_r_ was due to infectious diseases, particularly trichomoniasis. It has previously been reported that the majority of cases demonstrating lesions produced by *Trichomonas spp* affecting the oral cavity and choanal slit, have a poor prognosis [36], and our findings confirmed this. In this study, the unassisted mortality rate due to gunshot was 33.5%, greater than that reported by Richards et al, 2005 (14%) [37] or Ress and Guyer, 2004 (<20%) [38]. This is due to regional differences in firearms availability, hunting and legislation. In our work M_r_ had an approximate 30% value in the three most prevalent causes of trauma. Most of those cases suffered severe trauma with multiple body systems affected. Finally, the unassisted mortality rate found in our young orphaned group (18.9%) was similar to other reports (16.1%) [39].

According to the classification of clinical signs, M_r_ was over 50% when the nervous, respiratory or digestive systems were primarily affected or in cases of general systemic infectious or parasitic disease. The M_r_ was higher in birds with integument and musculoskeletal conditions. On the other hand, the higher M_r_ in animals apparently healthy on admission or with nutritional and metabolic conditions is suggestive of captivity-related complications and requires further investigation. In the authors' opinion, the present classification focusing on clinical signs allows a more accurate assessment of the rehabilitation protocols than those based on the primary cause of admission. Both classifications are useful; clinical classification allows a veterinary perspective, while the primary cause of admission allows an assessment of environmental causes and problems, and should be included in the analysis of dispositions of the rehabilitation of wild birds of prey.

The release rate in our study was higher in the orphaned young group, followed by fortuity and captive birds that were mainly affected by minor health conditions. The overall release rate of trauma cases was 24.3% (ranging from 1% of electrocution cases to 61.5% of birds suffering impacts with buildings). The release rates of gunshot, collision with vehicles and unknown trauma were very similar to those previously reported [14], [37], [38], being under 35% in all cases. On the other hand, the permanent captivity rate differs and needs special consideration. The final disposition of a non-releasable bird depends on the welfare and legal policies of the country or of the centre. Therefore, comparison of this rate could be useless if the rehabilitation criteria and policies are not specified. In our centre, euthanasia decision-making is based on welfare and economical criteria; thus the rate of permanently captive birds is relatively low.

Length of stay is a quality indicator parameter frequently used in human medicine [40]. In rehabilitation of wild raptors the decision of when to release an animal is based on the criteria related with the rehabilitation process (health status, fitness and behaviour), but also on external/ecological factors [41]. In fact, the longest periods of stay observed in birds admitted in winter and autumn were explained by the dates of the hunting season in the area of the study, as well as adverse weather conditions. Some migratory species such as *Circaetus gallicus, Pernis apivorus* and *Otus scops* were maintained at the centre until the next spring migration. As a general rule, the length of stay must be as short as possible in order to reduce the risk of captive-related complications, infectious and parasitic disease, and behavioural abnormalities [42]. The length of stay is thus a critical parameter in assessing the quality of rehabilitation protocols.

The parameter time to death provides direct insight into the initial assessment and prognostication, the overall rehabilitation process, as well as the validity of veterinary protocols. This complements understanding of the mortality and euthanasia rates. In all time dependent variables we have included the extreme values because they highlight the real daily work of the rehabilitation centre, with birds remaining in captivity for unknown reasons. Interestingly, the median time to euthanasia was 1 day. That means that the decision is taken at the moment of the admission, resulting in optimization of welfare and financial resources. On the other hand, the median time of death was 2 days even for the young orphaned group. This fact suggests that special care and a complete clinical evaluation should be performed on all young birds, despite their apparently healthy appearance.

In our work, we paid attention into the M_r_ and E_r_ over the P_90_ of the T_d_, as an indicator of undesirable or unexpected dispositions. The decision of euthanasia over 59 days was mostly taken due to complications related to trauma or musculoskeletal conditions. In our protocols, at 59 days most birds are in outside enclosures undergoing active flight conditioning. At this stage the decision to euthanize is taken in birds with musculoskeletal problems as well as those demonstrating abnormal behaviour incompatible with release to the wild.

Finally, a significant decrease in the unassisted mortality rate was observed in the traumatic category. This finding could be consequence of the improvement of both diagnostic and therapeutic protocols applied in the last years. The optimization of protocols for identifying specimens that are non-viable, has permitted the early euthanasia of these animals, avoiding unnecessary animal suffering and improving the management efficiency of resources.

In conclusion, the basic outcome research of the rehabilitation process of wild birds of prey and owls should include the four final disposition rates (Mr, Er, Rr and Cr), but also the parameters time until death (Td) and length of stay at the centre (Tr). The reports should also include the overall rates and the stratified analysis according to the cause of admission and the clinical entities. Moreover, both Td and Tr should be estimated by the overall group, but also stratifying by final decision and cause of admission and clinical entities. These six parameters are measurable items that should be considered as a baseline indicators for quality audits. Our results could represent a reference of a large amount of parameters related with the outcomes of the wildlife rehabilitation process that could be adapted by other centres as a start-point for further comparison. Finally, consensus of the professionals involved in rehabilitation of wild birds of prey is essential in order to develop evidence-based clinical guidelines and recommendations that will lead to an improvement of the rehabilitation procedure.
